# Adapting virtual agent interaction style with reinforcement learning to enhance affective engagement

**DOI:** 10.3389/fdgth.2025.1680605

**Published:** 2026-01-02

**Authors:** Christian Tamantini, Alessandro Umbrico, Francesca Fracasso, Gloria Beraldo, Gabriella Cortellessa, Andrea Orlandini

**Affiliations:** Institute of Cognitive Sciences and Technologies, National Research Council of Italy, Rome, Italy

**Keywords:** adaptive human-agent interaction, affective engagement, behavior change intervention, face emotion recognition, reinforcement learning

## Abstract

**Introduction:**

The ability of artificial agents to dynamically adapt their communication style is a key factor in sustaining engagement during human-agent interaction. This study introduces a reinforcement learning-based framework for real-time modulation of interaction style, aiming to maximize the affective valence of the user’s emotional response. The approach is domain-independent and designed for integration into scenarios where personalized and engaging dialogue is critical, such as in Behavior Change Interventions.

**Methods:**

To validate the system, we conducted a between-subjects user study involving N=20 participants, who completed a structured task, i.e. the URICA questionnaire, delivered either by an adaptive speech-based agent or a static screen-based interface. In the adaptive condition, the virtual agent employed Thompson Sampling to select between two communication styles (*enthusiastic* and *neutral*) based on real-time facial emotion recognition. The goal of the system was to reinforce the style that increased or maintained valence across successive interaction turns.

**Results:**

The reinforcement learning system successfully adapted its behavior based on individual users’ emotional feedback. Notably, a significant positive correlation was observed between users’ Psychoticism scores and the reinforcement of the *neutral* style (Spearman’s ρ=0.70, *p*-value = 0.04), indicating sensitivity to personality traits. Although no significant differences emerged in user-reported experience between conditions, this highlights that the adaptive speech-based agent preserved usability while successfully personalizing interaction based on affective cues.

**Discussion:**

These findings highlight the potential of adaptive agents to personalize interaction strategies in emotionally relevant contexts, even when the subjective user experience appears similar to that of static systems. The ability to align communicative behavior with user personality profiles supports the feasibility of deploying such models in long-term interventions, where maintaining user motivation and engagement is essential.

## 1 Introduction

Behavior Change Interventions (BCIs) aim to support individuals in achieving sustainable improvements in their habits and lifestyles, often as a supplement to conventional care (1). Digital and robotic systems have emerged as promising tools in BCIs, offering continuous engagement with users and assisting in progress monitoring and feedback delivery tasks (2). A fundamental step in BCI scenarios involves user profiling, typically performed through standardized questionnaires like, e.g., the *University of Rhode Island Change Assessment Scale* (URICA) (3), whose aim is to assess an individual’s readiness to change and informs the intervention strategy within the Transtheoretical Model (4, 5).

Artificial agents are increasingly employed to support the delivery of structured interactions, such as standardized questionnaires or coaching dialogues, in contexts ranging from healthcare to education and behavior change support (6). These systems offer the advantage of scalable deployment, allowing for interactive engagement while automating the collection of subjective and objective user data. Their use has been validated in various populations, including children (7) and individuals with autism spectrum disorders (8), showing promise in reducing clinician workload and enhancing user experience in repetitive or sensitive tasks.

However, the effectiveness of such agents in human-agent interaction is critically dependent on their ability to adapt to the user. A positive perception of the interaction plays a key role in promoting engagement, and tailoring the agent’s communicative behavior to match user preferences can substantially improve the quality and sustainability of the interaction (9). Previous research has explored the use of different interaction styles through variations in, e.g., language, facial expressions, and gestures (10). In some cases, agents have been endowed with context-dependent personality traits to better align with user expectations and task demands (11). Nonetheless, most existing approaches rely on static style definitions and evaluate their effect post hoc through standardized self-report measures, such as the Big Five Personality Test, without implementing real-time adaptation mechanisms.

Recently, a growing body of work has begun to investigate the use of Reinforcement Learning (RL) to support user-adaptive interaction policies (12). For example, RL has been used to personalize activity selection (13), medication reminders in elderly care (14), and long-term behavior shaping in task-driven dialogue systems (15). In some cases, social feedback signals have been integrated to adapt agent personality traits during interaction (16). However, these systems generally focus on task optimization rather than dynamically adjusting communication style to maximize affective quality or engagement. The lack of methods for real-time style adaptation limits the agent’s ability to respond empathetically and maintain user trust, especially in domains such as BCI, where engagement is critical to long-term adherence.

To address this gap, we introduce a general framework for affective adaptation in human–agent interaction that closes the loop between real-time emotion perception and online interaction style selection. The proposed approach uses a lightweight reinforcement learning mechanism to learn, during the interaction and for each individual user, which communicative style is more likely to support a positive affective trajectory. Rather than relying on predefined rules or manually engineered personalization strategies, the system infers the relation between style and affect through continuous updates driven by facial emotion recognition and a valence-based reward signal.

Although the framework is domain-independent and designed for flexible deployment across interactive settings, we evaluate it in a controlled and socially sensitive scenario representative of early-stage behavior change interventions. In this case study, the agent acts as a virtual coach guiding the user through an initial profiling session (17). This structured yet emotionally rich context allows the isolation and examination the dynamics of the adaptive mechanism, providing an interpretable testbed to assess its feasibility, responsiveness, and user-dependent learning patterns.

## 2 Materials and methods

**Figure 1:**
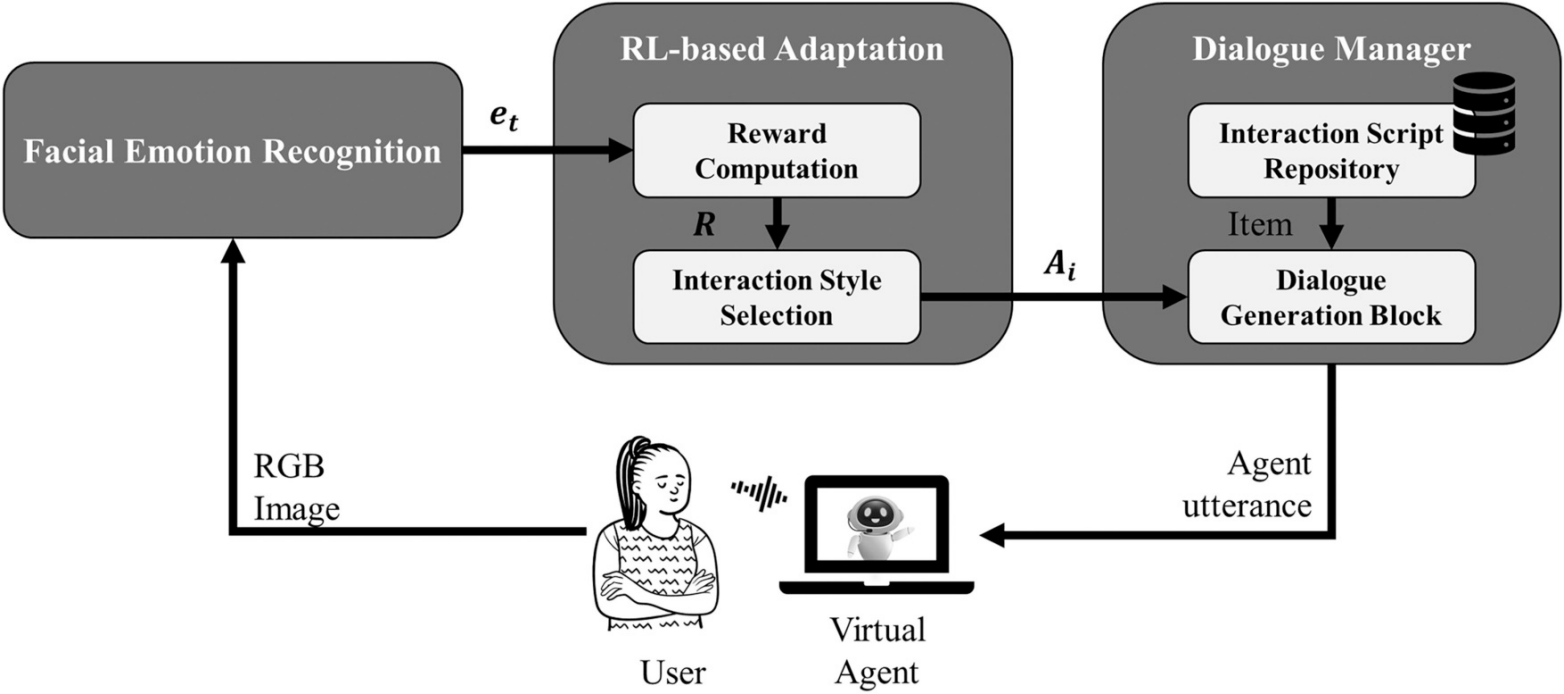
Overview of the proposed system architecture. The virtual agent observes the face of the user, it captures an RGB image to infer emotional states through the *Facial Emotion Recognition* module. This emotional signal ($e_{t}$) is further processed by the *RL-based Adaptation* module, which computes a reward (R) based on affective valence and selects an appropriate interaction style ($A_{i}$) for the next utterance. Then, the *Dialogue Manager* retrieves the corresponding item from a (pre-defined) *Interaction Script Repository* and passes it, along with the selected style, to the *Dialogue Generation Block*, which formulates the final utterance using a large language model. The generated utterance is then delivered to the user through a speech interface.

In this work, we propose a general architecture for adaptive virtual agent-based interaction, illustrated in Figure 1. The system is composed of three main modules: *Facial Emotion Recognition*, *Reinforcement Learning-based Adaptation*, and *Dialogue Manager*. These modules work in synergy to personalize the interaction in real time and optimize user engagement based on emotional feedback.

The *Facial Emotion Recognition* module continuously analyses the facial expressions of the user to estimate their emotional state during the interaction. This information is used as a proxy for affective engagement and serves as a basis for dynamically adjusting the agent’s communicative behavior.

The *Reinforcement Learning-based Adaptation* module is responsible for selecting the most appropriate interaction style at each turn. It is composed of two subcomponents: *Reward Computation*, which assigns a numerical value to the observed emotional responses based on their valence, and *Interaction Style Selection*, which determines whether the upcoming utterance should follow a *neutral* or *enthusiastic* communicative style. The adaptation mechanism is based on Thompson Sampling, allowing the agent to balance exploration and exploitation while optimizing the user experience over time.

The *Dialogue Manager* module manages the flow of verbal interaction. At each step, it retrieves the next item from the *Interaction Script Repository* and formats the corresponding utterance using a large language model, which rephrases the prompt according to the selected communication style. This ensures that the semantic content remains consistent while allowing the expression to adapt dynamically to the inferred emotional state of the user.

In this study, we define an interaction as the time interval between the moment the agent delivers an item to the user and the moment the user provides a response. During this interval, the system continuously monitors the facial expressions of the user, computes affective feedback, and determines the most suitable strategy for the next interaction turn.

### 2.1 Facial emotion recognition

The *Facial Emotion Recognition* module analyses the user’s facial expressions throughout the interaction to estimate their emotional state in real-time. The system employs a deep learning–based classifier that detects seven universally recognized basic emotions, i.e., *Angry*, *Disgust*, *Fear*, *Happy*, *Sad*, *Surprise* and *Neutral*, as defined in psychological research (18). These emotions are associated with distinct, cross-culturally identifiable facial patterns, making them a reliable basis for affective inference during interaction.

To support online adaptation, the module relies on a convolutional neural network trained on the widely used 2,013 facial expression dataset, which includes more than 35,000 labeled grayscale images captured in naturalistic, in-the-wild conditions (19). This choice provides greater robustness to spontaneous, non-posed expressions commonly observed during real interactions (20).

The network architecture follows a lightweight VGG-inspired design, consisting of six convolutional layers with Exponential Linear Unit (ELU) activations, batch normalization, max-pooling, and dropout for regularization (21). A dense layer with 128 units precedes the final softmax layer, which produces the probability distribution over the seven emotion classes. To improve generalization and reduce overfitting, the model was trained using extensive data augmentation (random rotations, zooms, translations, shears, and horizontal flips), a stratified 90/10 training–validation split, and the Adam optimizer with a learning rate scheduler and early stopping. The final model achieved an accuracy of 80.35% on the validation set.

During runtime, each frame from the camera (collected with a resolution of 1280×720) is processed through MediaPipe for face detection (22), cropped, resized to 48×48 pixels, converted to gray scale, and then fed into the CNN for inference. The resulting emotion predictions form the basis for valence estimation and subsequently drive the reinforcement learning–based adaptation mechanism.

### 2.2 Reinforcement learning-based adaptation

The *Reinforcement Learning-based Adaptation* module is responsible for dynamically adjusting the behavior of the agent to optimize the affective quality of the interaction. Its core function is to select, at each turn, the most suitable communication style based on the user’s emotional responses. By adapting the interaction in real time, the system aims to foster a more personalized and engaging user experience, with the overarching goal of sustaining attention and emotional involvement.

This module takes as input the sequence of facial expressions ($e_{t}$) detected during the current interaction turn and computes a reward signal that quantifies the emotional valence expressed by the user. The reward is calculated at the end of each turn as the average valence of the facial expressions observed during that interval. This scalar feedback is then used to update the agent’s behavior policy, allowing the system to reinforce the style ($A_{i}$) that most effectively promotes positive affect in subsequent turns.

#### 2.2.1 Reward computation

The reward signal quantifies the effectiveness of the agent’s communicative behavior based on the emotional responses of the user during the interaction. Each detected facial expression is associated with a valence score V(e) that reflects its affective polarity, as reported in Table 1. The scores are based on empirical values established in previous work (23), with higher scores assigned to positive emotions (e.g., *Happiness*), and negative emotions (e.g., *Sadness*, *Fear*) associated with lower values.

**Table 1:** Valence scores (V) assigned to each emotion (e) used in the reward computation.

Emotion (e)	Valence score (V(e))
*Happiness*	+0.76
*Surprise*	+0.40
*Neutral*	+0.00
*Anger*	−0.43
*Disgust*	−0.60
*Sadness*	−0.63
*Fear*	−0.64

The reward is calculated as in Equation 1 at the end of each interaction turn as the average valence of the emotional expressions observed during that time window:

$$R=\frac{1}{T}\cdot \sum_{t=1}^{T}V(e_{t}),$$ (1)

where $V(e_{t})$ is the valence score of the emotion detected at time t, and T is the total number of frames collected during the turn. This formulation captures the user’s overall affective experience during the interaction, reinforcing communicative styles that are associated with more positive emotional responses. By averaging over time, the system is encouraged to maintain emotionally engaging interactions while avoiding short-lived spikes or drops in affect.

Importantly, this reward formulation was selected to isolate the affective contribution of the agent’s communicative style while minimizing confounding effects due to momentary fluctuations or noise in the facial expression signal. Averaging valence across the entire response window provides a stable estimate of the emotional trajectory associated with each interaction turn, which is crucial in a reinforcement learning setting where the agent must rely on consistent feedback signals to update its policy.

Furthermore, focusing on variation of valence between consecutive turns enables the model to capture how the user’s affect evolves as a consequence of the agent’s behavior, rather than relying on absolute emotional states that may be influenced by individual baseline expressiveness. This makes the adaptation mechanism robust to inter-individual differences and allows the agent to modulate its style according to the dynamic, user-specific pattern of affective responses.

Although this reward formulation does not explicitly account for potential emotional differences intrinsic to the content of each questionnaire item, it provides a controlled and reliable signal for assessing the incremental effect of communicative style in a structured interaction.

#### 2.2.2 Interaction style selection

To adapt its communicative style in real time, the agent must decide at each interaction turn which style is most likely to produce a positive affective response. This can be formulated as a *multi-armed bandit* problem, where each communicative style represents an action with an initially unknown probability of success. As user reactions evolve, the system must balance the exploration of different styles with the exploitation of those that have already shown favorable outcomes.

To address this challenge, the proposed approach employs *Thompson Sampling*, a probabilistic bandit-based reinforcement learning algorithm widely adopted in adaptive interaction settings due to its data efficiency and robustness (24). Thompson Sampling maintains, for each style, a probability distribution representing the belief of the agent about its likelihood of success. At each turn, the algorithm draws a sample from each distribution and selects the style with the highest sampled value. After observing the user’s response, the algorithm updates the corresponding distribution, allowing interaction style selection to evolve dynamically during the interaction.

Each communicative style i is associated with a Beta distribution $\text{Beta}(\alpha_{i},\beta_{i})$, defined as reported in Equation 2:

$$\text{Beta}(x;\alpha ,\beta )=\frac{\Gamma (\alpha +\beta )}{\Gamma (\alpha )\Gamma (\beta )}x^{\alpha -1}(1-x{)}^{\beta -1},$$ (2)

where $\alpha_{i}$ and $\beta_{i}$ track the number of observed successes and failures, respectively, x∈[0,1], and Γ(⋅) is the Gamma function (25). All styles start with uniform priors ($\alpha_{i}=\beta_{i}=1$), ensuring equal initial selection probability. At each interaction turn, the algorithm samples, see Equation 3:

$$\theta_{i}∼\text{Beta}(\alpha_{i},\beta_{i}),$$ (3)

selects the style with the highest sampled value, and observes the resulting affective reward. Crucially, success is defined not by absolute valence but by its variation relative to the previous turn, using the formula reported in Equation 4:

$$\Delta R=R_{t}-R_{t-1}.$$ (4)

A non-negative ΔR increments $\alpha_{i}$ (success), whereas a negative ΔR increments $\beta_{i}$ (failure). At the first interaction turn, no previous reward is available; therefore, $R_{0}$ is initialized to zero, and the update rule is applied only from the second turn onward. This formulation reinforces styles that stabilize or improve the user’s affective state rather than maximizing positive expressions alone. The implemented Thompson Sampling for Interaction Style Selection approach is summarized in Algorithm 1.

Algorithm 1 **Thompson Sampling for Interaction Style Selection.**

1: **Input:** Number of styles *N*, prior parameters (*α*_*i*_, *β*_*i*_)

2: Initialize *R*_0_ ← 0     ▹ No prior affective information

3: Set *t* ← 1

4: **while** interacting with the user **do**

5:  Sample *θ*_*i*_∼Beta(*α*_*i*_, *β*_*i*_) for each style

6:  Select style *A* = arg max_*i*_ *θ*_*i*_

7:  Apply style *A* and compute reward *R*_*t*_

8:  **if** *t* > 1 **then**

9:   Compute Δ *R* = *R*_*t*_ − *R*_*t*-1_

10:   **if** Δ *R* ≥ **0 then**

11:     *α*_*A*_ ← *α*_*A*_ + 1          ▹Success

12:   **else**

13:     *β*_*A*_ ← *β*_*A*_ + 1        ▹Failure

14:   **end if**

15:  **end if**

16:  *t* ← *t* + 1

17: **end while**

Moreover, it is worth highlighting the differences of the proposed Thompson Sampling approach with respect to other different state-of-the-art reinforcement learning methodologies (26, 27). Unlike ε-greedy exploration, which introduces random exploratory actions that may be inefficient or redundant (12, 28), or Upper Confidence Bound (UCB) methods that require careful parameter tuning to balance exploration and exploitation (29), Thompson Sampling leverages Bayesian updating to naturally regulate exploration according to uncertainty. Compared with classical reinforcement learning methods such as Q-learning or policy gradient approaches, which often require extensive interaction data and tend to converge to rigid policies (30), Thompson Sampling is lightweight, adaptive, and well-suited to the short, sequential, and user-dependent nature of human–agent interactions.

The implemented *Interaction Style Selection* module enables the agent to update its communicative strategy in a principled and data-efficient way whenever new affective cues are observed. Once a style is selected for a given turn, this choice must be translated into an actual utterance that preserves the semantic content of the questionnaire while reflecting the desired communicative tone. This role is fulfilled by the *Dialogue Manager*, which operationalizes the selected style into the final system output delivered to the user.

### 2.3 Dialogue manager

The *Dialogue Manager* is responsible for orchestrating the verbal interaction with the user, ensuring both consistency in content delivery and adaptability in expressive style. At each interaction turn, it retrieves the next item from the *Interaction Script Repository*, i.e., a structured knowledge base that defines the sequence of prompts, questions, or topics the system is expected to deliver, and generates the corresponding system utterance according to the communication style selected by the *Interaction Style Selection* module.

Specifically, the Dialogue Manager receives two inputs at every step: (i) the current interaction style, chosen by the RL-based adaptation module based on recent user feedback, and (ii) the next item in the interaction script. The selected item is then passed to a large language model for utterance generation.

In this study, we operationalize two distinct communicative styles:

- The *neutral* style presents the interaction content using minimal, direct, and emotionally restrained phrasing. It is designed to convey the prompt in a straightforward manner, reducing cognitive and affective stimulation. This style may be preferable for users who value clarity, efficiency, or a more task-focused interaction.
- The *enthusiastic* style, by contrast, makes use of elaborated, motivational, and emotionally expressive language. It includes supportive cues and positive reinforcement aimed at stimulating user engagement and creating a more vivid social experience. Due to its expressive nature, this style is inherently more verbose, using more words to deliver the same informational content in a way that emphasizes warmth and encouragement. Importantly, in our implementation, the term *enthusiastic* refers to a linguistically richer and affectively positive mode of phrasing, rather than to prosodic animation or exaggerated emotional expressiveness.

To isolate the effect of verbal style alone, both styles are delivered using identical prosody, speech rate, and intonation. The adaptation is therefore restricted to the textual formulation of the utterance, ensuring that differences in user response are attributable solely to linguistic variation.

To implement these styles consistently and flexibly, the system employs GPT-4, accessed via OpenAI’s ChatGPT API, to generate the final system utterance. For each interaction turn, a structured prompt is dynamically composed to instruct the model to produce a response that reflects the selected communication style while preserving the original semantic content of the message. The prompt is formulated as follows:

*“Rephrase the following user-facing question in a [STYLE] manner, as defined below. Do not alter the meaning or structure of the item.*

*Style definition: [STYLE DEFINITION]*

*Question: ‘[ORIGINAL ITEM]’ ”*

In this format, the placeholder [STYLE] is replaced with either *neutral* or *enthusiastic*, depending on the output of the interaction style selection module. The prompt also includes a corresponding style definition to ensure consistent interpretation by the language model: the *neutral* style is defined as “concise, factual, and emotionally neutral,” while the *enthusiastic* style is described as “supportive, motivational, and positively expressive.” The placeholder [ORIGINAL ITEM] refers to the specific script item retrieved from the interaction repository and must remain semantically and structurally intact in the final output. This prompt formulation enables the system to dynamically tailor the style of its utterances without altering their informational content, ensuring standardized communication that can flexibly adapt to individual users. The explicit inclusion of style definitions minimizes ambiguity in the model’s stylistic rendering and supports reliable generation across turns.

**Figure 2:**
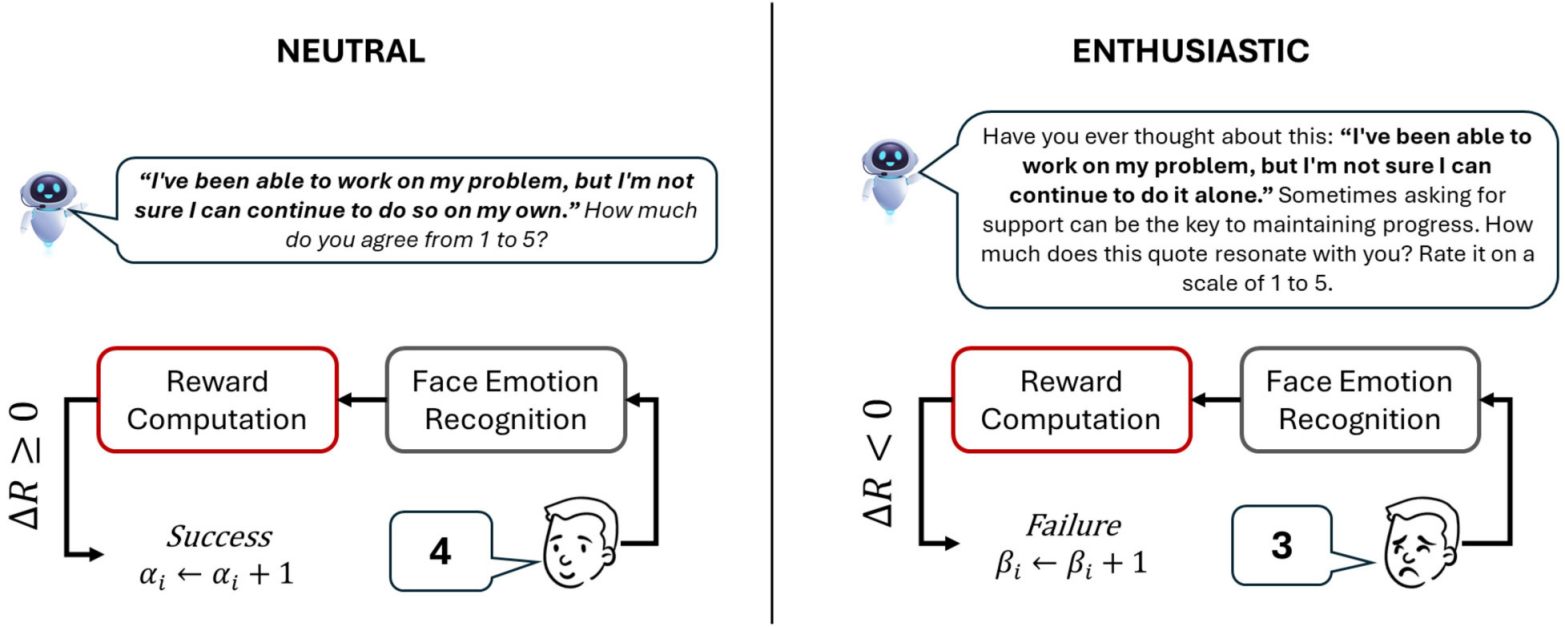
Illustration of two interaction turns expressed in either the *neutral* or *enthusiastic* style. After the virtual agent utterance, the user provides a numerical response to the URICA item and the facial expression is monitored. Only the detected facial emotion is processed by the *Face Emotion Recognition* module and used in the *Reward Computation*. The reinforcement signal is determined by the change in valence between consecutive turns (ΔR), which updates the Thompson Sampling parameters ($\alpha_{i}$) or ($\beta_{i}$) as a success (ΔR≥0) or failure (ΔR<0). The user’s numerical rating (e.g., 4 or 3) is not involved in the adaptation mechanism.

Figure 2 illustrates examples of how the same interaction script item is presented under the two communication styles. This design allows the system to adapt its verbal behavior in real time, modulating linguistic presentation in response to the emotional responses of the users.

### 2.4 Experimental validation

The primary goal of the experimental validation was to assess the ability of the system to learn and adapt its communication style to individual users, and to evaluate whether this adaptation enhances the quality of the interaction compared to a conventional questionnaire delivery method. Specifically, we aimed to observe how the reinforcement learning mechanism adjusted the behavior of the agent over successive interactions and whether these adjustments translated into a more engaging and emotionally resonant user experience.

To this end, we compared two clearly differentiated conditions for administering the URICA questionnaire. In the baseline (non-adaptive) Static Screen condition, participants completed the questionnaire through a standard Google Forms interface. No interaction style was implemented: the items were displayed in their original textual form, and participants selected their responses through a conventional linear graphical interface, with no speech, no adaptation, and no social interaction.

In the adaptive Speech-based condition, implementing the proposed RL-based adaptation strategy, the questionnaire was delivered by a virtual agent using a spoken dialogue interface. Only the verbal phrasing of each system utterance was adaptive: the RL module selected, at each turn, whether to express the item using the *neutral* or the *enthusiastic* communicative style. All other elements of the interaction were deliberately held constant. In particular, the prosody of the synthesized speech (voice, intonation, rhythm) and the appearance of the on-screen virtual avatar remained identical across turns, ensuring that any behavioural variation originated exclusively from the adaptive linguistic formulation.

The URICA questionnaire was selected due to its suitable length (32 items), which enables the observation of behavioral adaptation over extended interactions, and its established role in BCIs as a tool for assessing readiness for change. While the long-term goal of our research is to support the design of adaptive agents for behavior change coaching, the present study focuses on the initial user profiling phase (17). Ensuring a personalized and engaging interaction at this early stage is essential to foster user involvement and lay the groundwork for subsequent phases of tailored intervention.

#### 2.4.1 Experimental protocol

We recruited N=20 participants (10 males and 10 females, mean age 28.8±5.9). Participants were randomly assigned to either the adaptive speech-based or static screen-based condition, following a between-subjects design to avoid learning or carryover effects between modalities. This choice was not intended to elicit explicit user preferences between the two modalities, but rather to ensure that the adaptive behavior of the agent could be evaluated in a clean and unbiased manner. A within-subjects design would have exposed participants to both modalities, potentially shaping their facial expressiveness, response style, or expectations during the second exposure, thereby contaminating the reinforcement learning signal. By keeping the groups separate, we ensured that the system’s behavioral adaptation reflected the natural progression of the interaction rather than cross-condition influences. Each participant completed the URICA questionnaire using their assigned modality: either through a socially interactive system capable of adapting its communication style in real-time, or via a static digital form.

At the end of the session, all participants completed a set of standardized questionnaires aimed at profiling personality traits and evaluating their subjective experience. Specifically, the Eysenck Personality Questionnaire (EPQ) (31) was used to assess individual personality dimensions, and the Self-Assessment Manikin (SAM) (32) was administered to capture participants’ emotional responses to the interaction. Participants assigned to the adaptive speech-based condition were additionally asked to complete the Subjective Assessment of Speech System Interfaces (SASSI) (33), to evaluate their perception of the system’s vocal interface.

The EPQ consists of 100 binary (True/False) items distributed across four scales: Extraversion (range: 0–23), Psychoticism (0–32), Neuroticism (0–24), and Lie (0–21). These scales measure core personality traits: Extraversion captures sociability and outgoingness, Psychoticism reflects impulsivity and nonconformity, Neuroticism relates to emotional instability and vulnerability to stress, and the Lie scale estimates the tendency to respond in a socially desirable rather than truthful manner. The EPQ was included to explore potential associations between individual personality traits and preferences for different communicative styles, and to investigate whether personality dimensions modulate the effectiveness of the adaptive behavior of the system.

The SAM is a non-verbal pictorial questionnaire that evaluates the emotional state of the user along three independent dimensions: Valence (positive vs. negative emotional tone), Arousal (level of activation or excitement), and Dominance (sense of control over the situation). Higher ratings indicate greater levels of the respective emotional component. In this study, the SAM was administered to assess the emotional experience of the users during the interaction. The goal was to compare whether and how the interaction modality (adaptive speech-based vs. static screen-based) influenced users’ affective responses, providing insight into the emotional impact of dynamic adaptation in user profiling tasks.

In the adaptive speech-based condition, participants also completed the SASSI, a standardized tool designed to assess perceived usability and interaction quality in speech-driven systems. The questionnaire evaluates six key dimensions: Response Accuracy, Likability, Cognitive Demand, Annoyance, Habitability, and Speed. Each item is rated on a 5-point Likert scale (from 1 = strongly disagree to 5 = strongly agree), with higher scores reflecting more favorable user perceptions, except for Annoyance, where higher values indicate greater user frustration. The SASSI was employed to obtain a detailed evaluation of how users experienced the speech-based interaction in terms of usability, cognitive load, and overall satisfaction, providing complementary insights to the emotional and engagement measures collected through SAM.

In addition, the System Response Time (SRT), defined as the latency between the end of the user’s utterance and the start spoken reply of the system, was computed in the experimental sessions. This measure includes the processing times of the STT module, the generation of the system utterance through the GPT-based model, and the TTS synthesis, and was collected to quantify the responsiveness of the interactive loop. Furthermore, the potential influence of utterance length on processing delays was examined by computing Pearson correlations between utterance length and the duration of each processing step, using a significance level of 0.05.

#### 2.4.2 Experimental setup

The experiment was conducted on a Dell G5 personal computer equipped with a webcam (resolution 1280×720), stereo speakers, and digital-array microphones. The system was implemented in Python using the Robot Operating System (ROS) middleware. The experimental setup differed according to the assigned condition.

In the adaptive speech-based condition, participants interacted with a virtual agent that administered the URICA questionnaire using natural language. The system incorporated Speech-to-Text (STT) and Text-to-Speech (TTS) modules to support a spoken dialogue. While the current item was also displayed on screen to support comprehension, the interaction was designed to be speech-centric and socially engaging, with the communication style dynamically adapted by the agent at each turn.

In the static screen-based condition, participants completed the same questionnaire autonomously using a standard web interface implemented in Google Forms. No speech interaction or stylistic adaptation was provided, replicating a traditional self-administered format.

To enable real-time adaptation based on users’ emotional feedback, the system integrated the *Facial Emotion Recognition* module, as described in Section 2.1. In parallel, STT modules transcribed user responses to ensure numerical validity; invalid responses triggered a polite reprompt. TTS was used to synthesize the agent’s utterances. While prosody and intonation were kept consistent across conditions, the verbal phrasing varied according to the communication style selected by the adaptation policy, enabling a dynamically personalized user experience.

**Figure 3:**
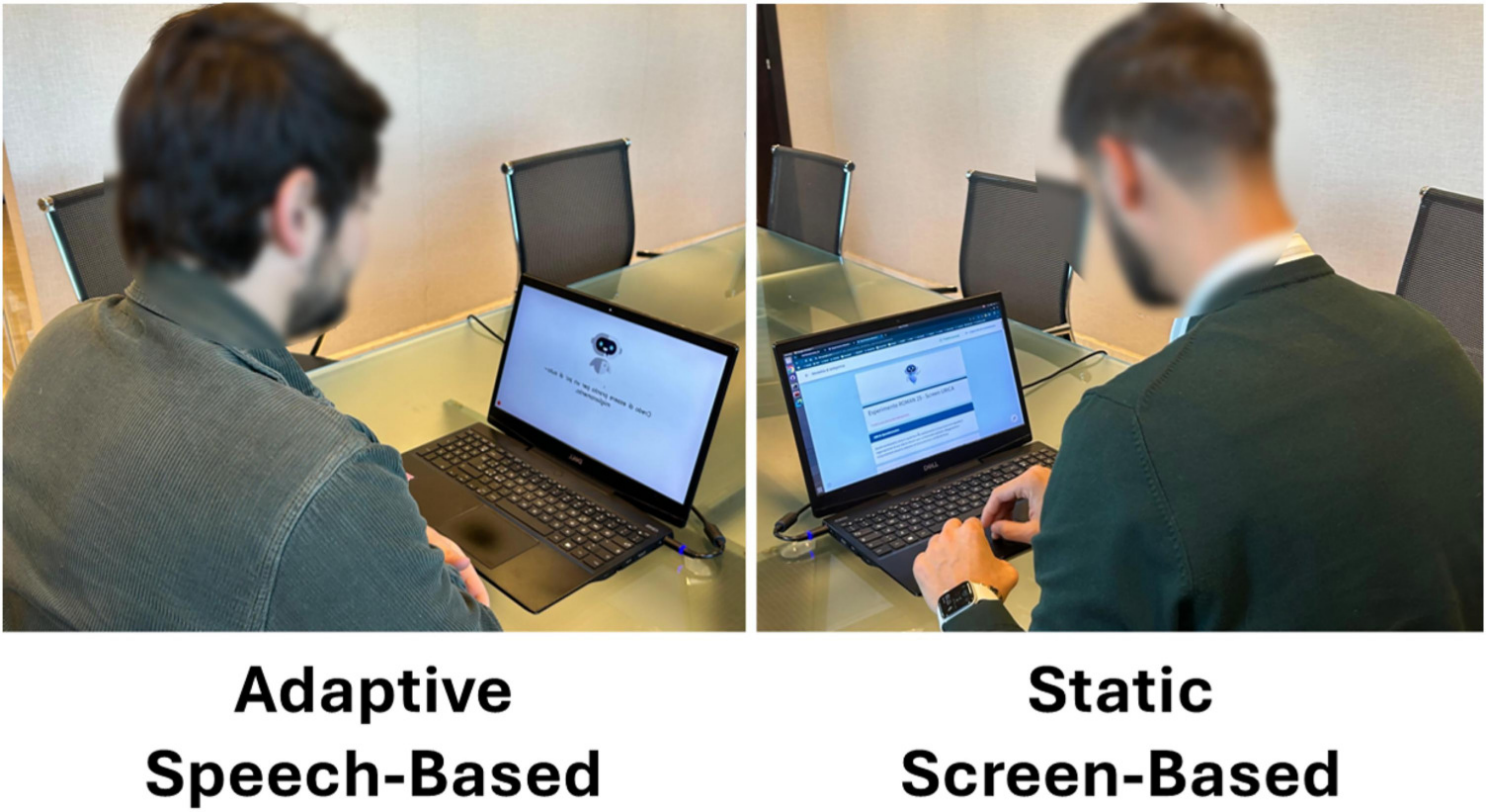
Experimental setup illustrating the two conditions. In the adaptive speech-based condition, the participant interacts vocally with the system, which adapts its communication style in real time. In the static screen-based condition, the participant completes the questionnaire using a standard digital interface without speech or adaptation.

A photo of two representative participants during the experimental session is shown in Figure 3.

### 2.5 Evaluation metrics and statistical analysis

The effectiveness of the proposed system was evaluated through analyses of both its learning behavior and the perceived quality of the user experience.

To assess how the reinforcement learning mechanism adapted to individual users, we analyzed data from participants in the adaptive speech-based condition only. Specifically, we examined the evolution of the α values associated with the *enthusiastic* and *neutral* interaction styles across interaction turns, as well as their final values at the end of the session. These values reflect the cumulative number of positively reinforced interactions, providing insight into the system’s behavioral preferences over time and its ability to personalize communication.

Additionally, for participants in the adaptive speech-based group, we investigated whether individual personality traits influenced the RL model’s behavior. To this end, we computed Spearman’s rank correlation coefficients between the final α values for each interaction style and the scores from the EPQ (Eysenck Personality Questionnaire). Spearman’s method was chosen due to its robustness against non-normal distributions. Statistical significance was assessed using a two-tailed test with a threshold of p<0.05.

To compare affective user experience across interaction modalities, we conducted a Mann-Whitney U test on the SAM (Self-Assessment Manikin) scores, including all participants from both conditions (adaptive speech-based and static screen-based). This non-parametric test was used to evaluate potential differences in perceived emotional valence, arousal, and dominance. Its distribution-free nature makes it suitable for analyzing subjective ratings. All statistical analyses were performed with a significance threshold of p<0.05.

## 3 Results and discussions

**Figure 4:**
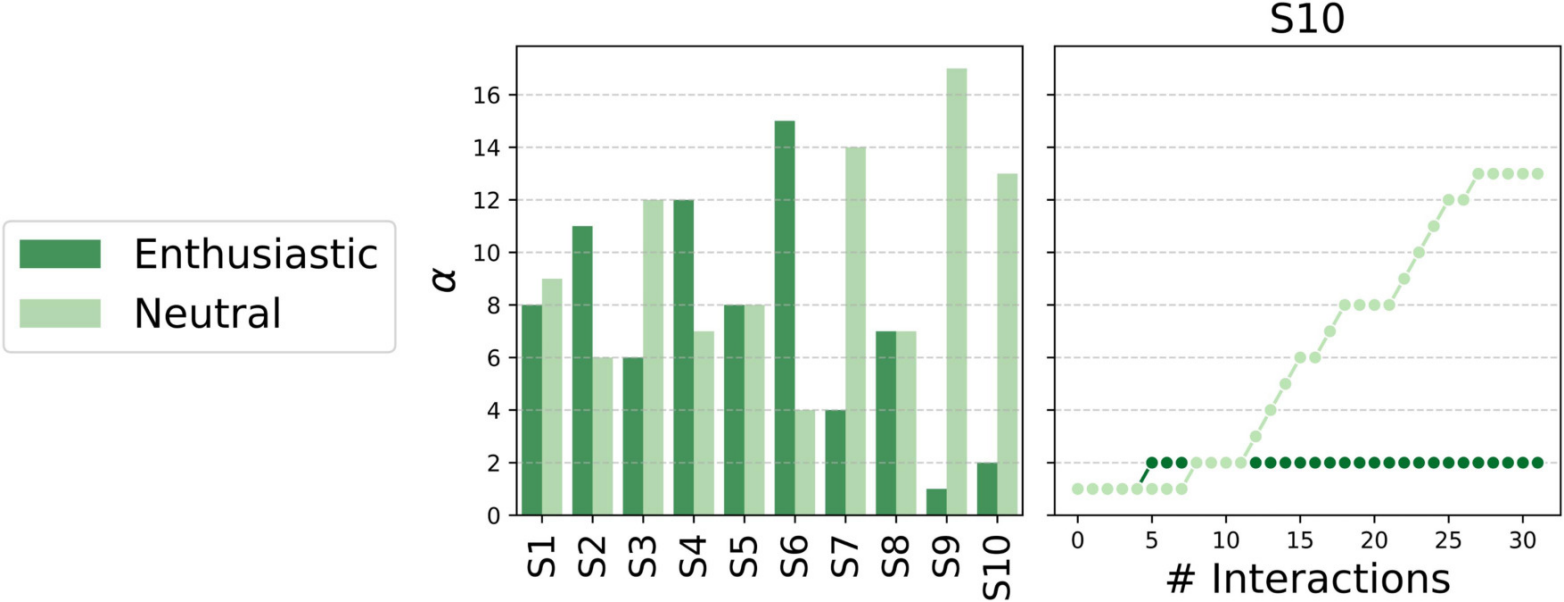
Overview of the reinforcement learning adaptation process. The left panel shows the final α values for extravert and introvert interaction styles across all Vocal participants, highlighting individual differences in the learned preference. The right panel illustrates the evolution of α values throughout the interaction for a representative participant, demonstrating how the system progressively converges toward a dominant interaction style.

The ability of the proposed reinforcement learning (RL) system to tailor its interaction style to individual users was evaluated by analyzing the number of successful interactions, quantified by the final α values assigned to the *neutral* and *enthusiastic* styles. Figure 4(left) presents the distribution of α values across all participants in the adaptive speech-based condition. These results highlight substantial inter-individual variability in the learned communication strategy, suggesting that the system did not converge toward a single dominant style but instead adapted dynamically to each user’s emotional responses.

To illustrate the learning dynamics over time, Figure 4(right) reports the evolution of α values for a representative participant (S10). The system progressively reinforced one style over the other as the interaction unfolded, reflecting its capacity to update behavioral choices based on the valence of the user’s facial expressions. This trend demonstrates the RL model’s ability to learn from interaction feedback rather than relying on a static or predefined policy.

A closer analysis of the final α distributions revealed that the system converged toward the *enthusiastic* style for 3 participants (S2, S4, S6), while for 4 others, the *neutral* style consistently led to more successful interactions (S3, S7, S9, S10). For the remaining 3 participants (S1, S5, S8), no clear preference emerged, with comparable α values for both styles at the end of the session. This outcome suggests that, in most cases, the system was able to identify and reinforce the interaction strategy that elicited more positive emotional responses from the user.

The absence of a dominant style in a subset of participants may reflect balanced emotional feedback across both communication styles or insufficient differentiation to drive strong reinforcement. Notably, this variability does not indicate a failure of the adaptation mechanism but rather underscores its flexibility: the system does not enforce a uniform strategy but instead continuously adjusts its behavior to align with user-specific feedback patterns. This dynamic and personalized adaptation is a key feature of the proposed model, enabling more nuanced and user-sensitive interactions.

To explore potential associations between the behavior of the reinforcement learning mechanism and individual user characteristics, we conducted correlation analyses between participants’ personality traits, measured using the EPQ, and the final α values assigned to the *neutral* and *enthusiastic* interaction styles. Table 2 presents the resulting Spearman correlation coefficients (ρ) and their corresponding *p*-values for each EPQ trait, indicating the strength and significance of the relationship between personality dimensions and the system’s learned preferences.

**Table 2:** Spearman correlation coefficients (ρ) and *p*-values between the personality traits from EPQ and the final α values for each interaction style.

EPQ Scale	$\alpha_{\text{enthusiastic}}$		$\alpha_{\text{neutral}}$	
	ρ	*p*-value	ρ	*p*-value
Extraversion	−0.03	0.94	−0.17	0.66
Psychoticism	−0.74	**0.02**	0.70	**0.04**
Neuroticism	0.08	0.83	−0.23	0.55
Lie	0.16	0.69	−0.10	0.80

Significant correlations (*p*-value <0.05) are marked in bold.

A strong and statistically significant negative correlation was found between Psychoticism and $\alpha_{\text{enthusiastic}}$ (ρ=−0.74, *p*-value = 0.02), alongside a positive correlation with $\alpha_{\text{neutral}}$ (ρ=0.70, *p*-value = 0.04). This suggests that individuals with higher levels of Psychoticism were more responsive to the *neutral* interaction style. This result is consistent with psychological literature, which associates high Psychoticism with reduced social engagement and a lower sensitivity to emotionally expressive communication (34). In adapting to these users, the system appears to have reinforced a more restrained and predictable interaction style, likely because neutrally phrased utterances elicited more stable or positive valence in this subgroup. While we did not measure explicit user preferences, one plausible interpretation is that the *neutral* style, being shorter and less expressive, may have reduced the cognitive load associated with the task. This suggests that the system’s behavior reflects an implicit sensitivity to the ease with which different individuals process and react to linguistic input. Such results illustrate the capability of the system to tune its behavior according to individual socio-emotional tendencies and to adapt its communicative style in ways that align with user-specific affective patterns.

No significant correlations emerged for Extraversion, Neuroticism, or Lie, indicating that the system’s adaptive behavior was not strongly associated with these traits. One might have anticipated a behavior-matching effect, for instance, that more extraverted users would prefer an *enthusiastic* style, yet the non-significant correlation for Extraversion suggests otherwise. This observation aligns with prior human-robot interaction studies showing that interaction style preferences can be task-specific, and that users may sometimes favor contrasting rather than congruent styles (35). In certain contexts, a communicative agent that counterbalances rather than mirrors the user’s disposition may be perceived as more effective or supportive, thereby attenuating the expected alignment between personality profiles and interaction preferences.

**Figure 5:**
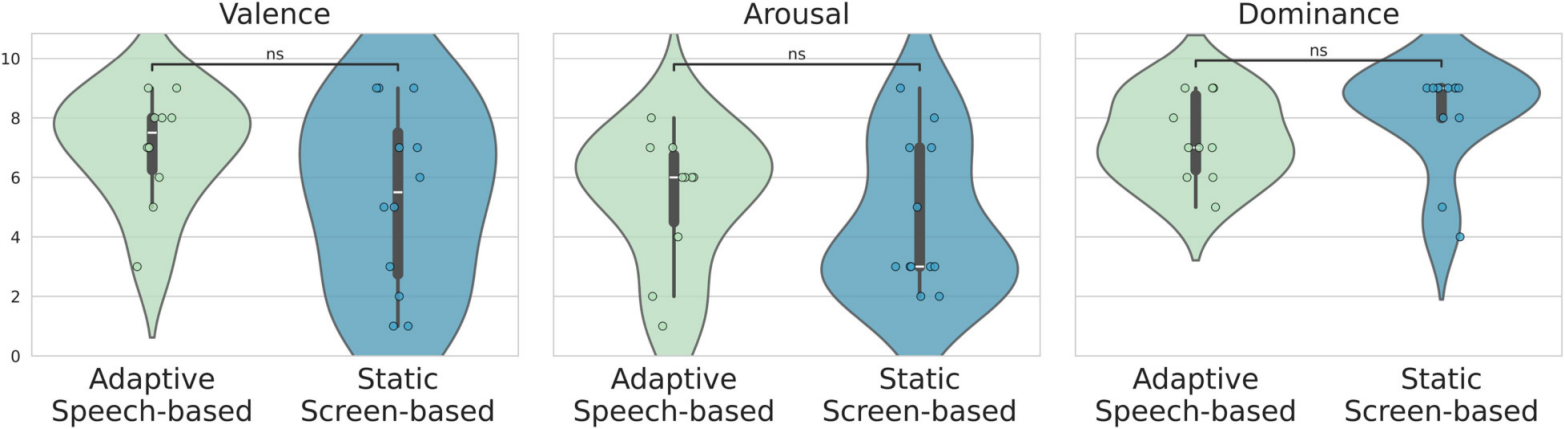
Comparison of user experience ratings between the Vocal and Screen conditions using the Self-Assessment Manikin scales. The violin plots illustrate the distribution of reported Valence, Arousal, and Dominance. Higher Valence scores indicate a more positive experience, higher Arousal scores reflect greater engagement, and higher Dominance scores suggest a stronger sense of control during the interaction.

To evaluate the perceived quality of the interaction across conditions, we analyzed participants’ responses to the Self-Assessment Manikin (SAM) questionnaire, which measures emotional experience along three dimensions: Valence, Arousal, and Dominance. As shown in Figure 5, participants in both the adaptive speech-based and static screen-based conditions reported comparable emotional responses across all dimensions.

Valence scores, reflecting the pleasantness of the interaction, were slightly higher, on average, in the adaptive speech-based condition, suggesting a trend toward a more positively perceived experience. However, the difference was not statistically significant (*p*-value = 0.3). Arousal scores, indicating the level of emotional activation, were similarly distributed across conditions, implying that both interaction modalities were comparably engaging. Finally, Dominance scores, which reflect the participant’s perceived control during the interaction, also showed no significant difference (*p*-value = 0.2).

Overall, these results suggest that while the adaptive speech-based system may have offered a slightly more positive experience, both modalities elicited similar emotional responses, supporting the usability of both adaptive and static approaches in questionnaire-based interactions. Crucially, the absence of statistically significant differences should not be seen as a lack of benefit from adaptation. The between-subjects design meant that each participant experienced only one modality, preventing direct comparison and associated bias. As such, their evaluations reflect a direct and unbiased perception of the interaction. The comparable ratings across conditions indicate that, in this structured scenario, the adaptive speech-based agent did not produce a noticeable change in perceived interaction quality relative to the static questionnaire. Importantly, this does not imply that the adaptive mechanism is unnecessary; rather, it shows that the introduction of an adaptive, speech-based virtual agent does not introduce additional cognitive or emotional burden compared to a traditional GUI. Given that GUI-based questionnaires can become repetitive or effortful during prolonged use (36, 37), the ability of an adaptive agent to maintain comparable subjective experience while supporting dynamic linguistic adaptation may become particularly valuable in longer or more naturalistic interactions. This reinforces the idea that adaptive systems can be integrated into user-facing applications without compromising perceived usability, while still offering the advantage of personalization.

Results from the SASSI questionnaire revealed an overall positive perception of the adaptive speech-based system. The participants rated the interaction as highly likable (4.79±0.42) and reasonably accurate in recognizing their responses (3.28±0.19). The cognitive demand was rated at 3.66±0.53, suggesting that while the interaction required a moderate level of mental effort, it was not perceived as burdensome. Importantly, annoyance levels remained low (2.00±0.61), indicating that the system was not found to be frustrating or irritating.

By contrast, ratings for habitability (2.31±0.53) and speed (2.96±0.49) were slightly lower. These dimensions reflect how naturally the system could be interacted with and how quickly it responded. The lower scores suggest potential areas for improvement, particularly in refining the dialogue flow and enhancing responsiveness to promote a more seamless user experience.

To assess the responsiveness of the adaptive speech-based system and verify that turn-taking delays did not interfere with users’ affective reactions, the SRT was computed for each interaction turn. Across all interaction turns in the adaptive condition, the average SRT was 4.11±0.67 s. Breaking the total latency into its constituent components, the STT step required on average 1.60±1.81 s, GPT-based utterance generation required 1.09±0.25 s, and TTS synthesis required 2.01±0.45 s. To assess whether processing delays were driven by linguistic content, we examined correlations between utterance length and the duration of each processing step. STT time showed only a weak, non-significant association with user utterance length (ρ=0.34, p−value=0.28). GPT generation time increased moderately with the length of the system-produced text (ρ=0.69, p−value=0.01)), and TTS synthesis time showed a strong correlation with utterance length (ρ=0.90, p−value<0.01)), as expected. It is therefore clear that the majority of the computation time is spent by the system in verbalizing the content generated by the LLM, in a manner that is strongly dependent on the length of the text to be verbalized.

Overall, the observed latencies fall within a range that is consistent with typical delays in cloud-based conversational systems and were not sufficiently long to produce noticeable disruptions in the interaction flow (38, 39). Importantly, the variance in SRT across turns remained limited, suggesting that fluctuations in system responsiveness are unlikely to have introduced systematic noise into the affective reward estimates used by the RL algorithm.

Overall, the results suggest that the adaptive speech-based interaction, despite its dynamic adaptation to user feedback, did not significantly alter the participants’ subjective experience compared to the static screen-based interface. Nonetheless, the trend toward higher Valence scores in the adaptive condition indicates that refinements to the reinforcement learning model and dialogue generation strategies could further enhance the perceived quality of the interaction. Additionally, responses to the SASSI questionnaire confirmed the general acceptability of the speech-based system: participants reported high likability and low annoyance, though some limitations were highlighted in terms of speed and habitability, pointing to opportunities for improving the naturalness and responsiveness of the dialogue.

These findings indicate that, within this structured and highly constrained questionnaire setting, the adaptive system was perceived as just as positive and acceptable as a simpler static interface. This equivalence is meaningful: it demonstrates that introducing a reinforcement learning mechanism that adapts linguistic behaviour in real time does not impose additional cognitive or emotional burden on users. At the same time, the adaptive framework offers capabilities that a static questionnaire cannot provide, such as learning online which communicative style is more supportive for a specific user and adjusting its behaviour accordingly.

Importantly, the observed convergence patterns in the Thompson Sampling parameters and their association with personality traits show that the system did not simply behave as a scripted agent but discovered user-dependent style–affect relationships directly from the interaction data. While the controlled nature of the task limits the emergence of measurable subjective benefits at the group level, these individual learning patterns illustrate the potential of the proposed framework to personalise interaction in a data-driven manner.

These strengths are expected to become more relevant in richer, longer, or more naturalistic scenarios, typical of behaviour change interventions, where sustaining engagement over time is essential and where adaptive, affect-aware interaction can offer advantages that do not emerge in short, highly structured tasks such as the one used here.

### 3.1 Limitations

The present findings should be interpreted in light of some limitations related to the experimental design and the affective modelling approach implemented in this study.

First, the use of a between-subjects design with two non-overlapping groups (10 participants per condition) was necessary to preserve the integrity of the reinforcement learning signal and to avoid carryover effects that could bias users’ emotional expressions across modalities. However, this choice limits the generalizability of the results and prevents direct assessment of individual preferences for one modality over the other. Personalization was therefore evaluated indirectly through the adaptive behavior of the system and its associations with personality traits, rather than through explicit comparative judgments and/or alignment with user preferences.

A second limitation concerns the controlled nature of the interaction scenario. Administering a structured questionnaire provides a stable and repeatable context for observing the learning dynamics of the adaptation mechanism, but it also constrains the naturalness and spontaneity of users’ emotional expressions. The restricted interaction space may have reduced the variability of affective cues and limited the breadth of behaviors to which the agent could adapt. Richer, open-ended, or ecologically naturalistic scenarios would likely elicit a wider range of emotional responses and provide a more informative test bench for adaptive behavior.

Further limitations relate to the affective sensing methodology implemented in this experimental scenario and reward formulation. The reinforcement learning mechanism presented in this work relied exclusively on changes in facial valence between consecutive interaction turns, under the assumption that increases or stability in valence reflect an improved engagement. While valence dynamics offer a practical real-time cue, they capture only a portion of the multidimensional construct of engagement. Cognitive effort, attention, and motivational involvement are not directly represented, and positive facial expressions, such as smiles, may not always correspond to genuine interest or preference. Additionally, although the facial emotion recognition module showed solid performance on publicly available datasets, vision-based systems remain sensitive to environmental factors such as lighting, head pose, and occlusions, which may reduce reliability in unconstrained settings (40). Facial cues alone may not fully reflect internal emotional states, particularly when affect is subtle or deliberately suppressed (41). Future implementations could benefit from integrating complementary modalities, such as speech prosody, heart rate variability, or electrodermal activity, to obtain a more comprehensive and multimodal estimate of user engagement (42, 43). Lastly, the reward function did not account for the intrinsic emotional load of the questionnaire items themselves. Different URICA items may naturally evoke more positive or negative affect independently of the communicative style implemented by the virtual agent, yet the current model does not incorporate topic-level weighting or semantic analysis to control for this factor. Anyhow, this simplified formulation was intentionally adopted to isolate the incremental effect of the agent behavior in a controlled and repeatable setting, but future work should examine how item semantics interact with emotional responses, particularly in more ecologically valid or in-the-wild interactions where content variability is intrinsically higher.

Taken together, these limitations highlight both the promise and the challenges of using reinforcement learning for adaptive human–agent interaction, underscoring the need for broader, multimodal, and more ecologically grounded evaluations in future studies.

## 4 Conclusion

This study presented a general framework for enhancing affective engagement in human–agent interaction, designed to close the loop between real-time emotion perception and adaptive behaviour. The proposed system combines facial emotion recognition, a valence-based reward signal, and a lightweight reinforcement learning mechanism to learn online which communicative style is more supportive for each individual user. Unlike approaches based on manually defined rules or static dialogue strategies, the framework enables the agent to infer style–affect relationships directly from interaction data, offering a flexible and domain-independent foundation for personalized interaction.

The controlled experimental setting used to validate the proposed solution provided a stable test bench for isolating and assessing the dynamics of the adaptation process. The results demonstrate that the system is capable of adjusting its behaviour at an individual level: the heterogeneous accumulation of α values across users indicates that the model selectively reinforced either the *enthusiastic* or *neutral* style depending on the affective trajectories observed during the interaction. The significant association between Psychoticism scores and reinforcement of the *neutral* style further supports the system’s sensitivity to inter-individual socio-emotional differences, despite the simplicity of the task.

In terms of user experience, no statistically significant differences emerged between the adaptive speech-based and static screen-based conditions in self-reported emotional states. However, the adaptive condition showed a mild trend toward higher Valence, and SASSI ratings confirmed high overall acceptability, with users reporting low annoyance and high likability. Importantly, the adaptive system achieved these outcomes without imposing additional cognitive or emotional burden, despite its richer and dynamically evolving behaviour.

Overall, while the present structured questionnaire task may not fully reveal the measurable advantages of adaptation at the group level, the findings show that the proposed framework is both viable and capable of capturing meaningful inter-individual variability. This establishes a solid foundation for deploying affect-aware adaptive mechanisms in richer, longer, and more ecologically valid scenarios, where sustaining user engagement and tailoring communication to evolving emotional states are essential for promoting long-term adherence and success.

Future work will address these limitations by increasing the sample size, exploring longitudinal and more naturalistic interactions, and testing experimental designs that better balance ecological validity with experimental control. In parallel, from a technological perspective, future efforts will focus on enhancing the RL model by incorporating additional user signals, such as speech prosody and physiological data, and by evaluating the system across richer interaction contexts.

## Data Availability

The raw data supporting the conclusions of this article will be made available by the authors, without undue reservation.
